# A Novel Prediction Tool Based on Large Cohorts to Determine the Cancer-Specific Survival Probability of Patients With Locally Advanced Pancreatic Cancer After Irreversible Electroporation Treatment

**DOI:** 10.3389/fonc.2020.00952

**Published:** 2020-06-30

**Authors:** Chaobin He, Xin Huang, Yu Zhang, Xiaojun Lin, Shengping Li

**Affiliations:** ^1^State Key Laboratory of Oncology in South China, Department of Pancreatobiliary Surgery, Collaborative Innovation Center for Cancer Medicine, Sun Yat-sen University Cancer Center, Guangzhou, China; ^2^State Key Laboratory of Ophthalmology, Zhongshan Ophthalmic Center, Sun Yat-sen University, Guangzhou, China

**Keywords:** locally advanced pancreatic cancer, irreversible electroporation, cancer-specific survival, nomogram, prognosis

## Abstract

Irreversible electroporation (IRE) is a novel method which was especially suitable for the treatment of locally advanced pancreatic cancer (LAPC). The purpose of this study was to evaluate probabilities of overall survival (OS) and cancer-specific survival (CSS) in patients with LAPC after IRE treatment and to construct nomograms to predict survival for these patients. Data of patients were retrospectively collected from the Surveillance, Epidemiology, and End Results (SEER) database and medical records of Sun Yat-sen University Cancer Center (SYSUCC). A total of 312 LAPC patients after IRE treatment were included into this study. The 3-year cumulative incidence of cancer-specific mortality for patients with LAPC after IRE treatment was 74.3%. Nomograms for predicting probabilities of OS, CSS, and non-cancer-specific survival (NCSS) were built and calibrated with the concordance index (C-index) and the area under receiver operating characteristic (ROC) curve (AUC). The established nomograms were well-calibrated, with C-indexes of 0.782 for OS prediction, 0.729 for CSS prediction, and 0.730 for NCSS prediction. Compared with the TNM stage system, the established nomograms displayed higher values of AUC and showed better discriminatory power for predicting OS, CSS, and NCSS. These nomograms were well-calibrated and could serve to guide management of LAPC patients after IRE treatment.

## Introduction

Pancreatic cancer (PC) is the second most common gastrointestinal malignancy and the fourth leading cause of cancer death worldwide (1). As one of the most lethal and challenging malignancies, PC has a dismal prognosis with a 5-year survival rate of only 8% (2). Surgical resection remains the only curative modality for patients with PC. However, most of the patients were diagnosed at advanced stages and the surgical resection rate was only 20% (3, 4) because of metastasis (40%) or involvement of major vascular structures (40%), such as celiac artery, hepatic artery, superior mesenteric artery, and other structures of the portovenous axis (5). Locally advanced pancreatic cancer (LAPC) evolves without evidence of distant metastasis and, on macroscopic level, is represented with surrounding vascular involvement (6). A typical treatment which begins with system therapy to control micrometastatic diseases followed by radiation for local control may provide the best benefit for these patients. Previously, the management of LAPC patients foresees the use of gemcitabine based on chemotherapy in association or not with radiotherapy, achieving marginal benefits in terms of elevating overall survival. Moreover, such multimodality therapy can only downstage a small proportion of patients to resectable diseases. Although FOLFIRINOX (5-fluorouracil, leucovorin, irinotecan, and oxaliplatin) are used most recently as neoadjuvant setting for LAPC and have achieved improved survival, majority of LAPC patients remain ineligible for curative intent of surgical resection (6). In addition, patients with unresectable LAPC indeed own a poor median survival of only 6–11.5 months in most of prospective clinical trials despite advances in chemotherapy, radiotherapy, and chemoradiotherapy (7, 8). In this term, apart from systemic chemotherapy and radiation therapy, a new therapy method should be explored to improve the prognosis of LAPC patients.

Patients with local advanced, albeit unresectable PC should theoretically benefit from maximal local therapy. As a novel ablative procedure, irreversible electroporation (IRE) is a potential solution for the treatment of LAPC, which was widely performed intraoperatively (9), laparoscopically (10), or percutaneously (11) since 2009 (12). Instead of causing a thermal-based coagulative necrosis, IRE induces permanent cell membrane porosity by high-voltage and microsecond-length pulses, which causes permanent cell death without the destruction of the nearby structure (13–15). This unique feature of procedure makes IRE an ideal palliative treatment for LAPC patients, with surprisingly prolonged survival (16). With more and more use of this treatment, it is necessary to identify the clinical and pathological features of LAPC patients who received IRE treatment. Moreover, the most frequently used stage system, the 8th edition Tumor-Node-Metastasis (TNM) stage system of the American Joint Commission on Cancer (AJCC) (17), only focuses some of the pathological factors, ignoring some other potential variables, such as age and tumor grade. It is believed that the individual prognostic stage based on personal evaluation of prognostic factors is more helpful for personalized treatment. Therefore, it is necessary to establish specialized prognostic-stage systems to stratify the prognosis of LAPC patients after IRE treatment.

In addition, most of the included patients are diagnosed at their old ages. The negative impacts of the increasing ages on organ function and the varieties of age-related comorbidities, which are considered as competing risk events, may dilute or negate the benefit of the treatment (18). Therefore, it is important to consider the competing risks when evaluating prognosis. However, to our knowledge, there are no prognostic-stage systems considering both overall survival (OS) and cancer-specific survival (CSS) for LAPC patients after IRE on the basis of population-based data.

In the present study, survival exploration of LAPC patients based on OS analysis and competing risk analyses was conducted with the Surveillance, Epidemiology, and End Results (SEER) database, and the nomograms were established to estimate rates of OS and CSS for LAPC patients after IRE treatment.

## Patients and Methods

### Patients

Data of LAPC patients after IRE were extracted from the SEER database from 2012 to 2015, using the SEER^*^Stat software version 8.3.5. The study cohort consisted of patients with the following International Classification of Diseases for Oncology, Third Edition (ICD-O-3), histology codes 8010/3, 8021/3, 8140/3, 8255/3, and 8263/3, and the ICD-O-3 site codes C25.1, C25.2, C25.3, and C25.8. The following patients were excluded from this study: (1) patients with second primary cancer; (2) patients who were younger than 18 years; (3) patients not radiologically and pathologically diagnosed of LAPC; (4) patients who had received treatments other than IRE, including resection and radiotherapy; (5) patients whose information of survival, follow-up, or other factors were incomplete; and (6) patients with distant metastases or those whose tumor was not classified as T4 stage. Patients were randomly selected to serve as the training and internal validation cohorts in a ratio of 2:1. The second cohort of patients was from the Sun Yat-sen University Cancer Center (SYSUCC) (2015–2019). The exclusion criteria were the same as in our previous study (19). The patients from the SYSUCC database were used as the external validation cohort.

### Data Collection

The clinical variables, such as age, gender, and chemotherapy, and clinical and pathological variables of patients, including tumor site, grade, size, TNM stage, follow-up information, and cause of death, were extracted from the SEER database and SYSUCC database. Seventy years was used as the cutoff value for age at diagnosis. OS, CSS, and non-cancer-specific survival (NCSS) were defined as the duration from the date of treatment to death due to all causes, cancer and other causes, and last follow-up, respectively.

### Statistical Analysis

The probability of cancer-specific and non-cancer-specific death was evaluated by the cumulative incidence function (CIF) and compared by Gray's test (20). OS was analyzed using the Kaplan–Meier method and compared by the log-rank test. The hazard ratio (HR) and the associated 95% confidence interval (CI) were calculated. The Fine and Gray's model (21) and Cox regression model were used to build the competing risk nomograms and the nomogram for predicting OS. The established nomograms were calibrated with calibration curves and evaluated by concordance index (C-index) and the area under ROC curves (AUC) (22, 23).

R version 3.4.2 software (The R Foundation for Statistical Computing, Vienna, Austria, http://www.r-project.org) was used for statistical analyses. A two-tailed *P* < 0.05 was considered statistically significant.

## Results

### Patient Characteristics

**Figure 1:**
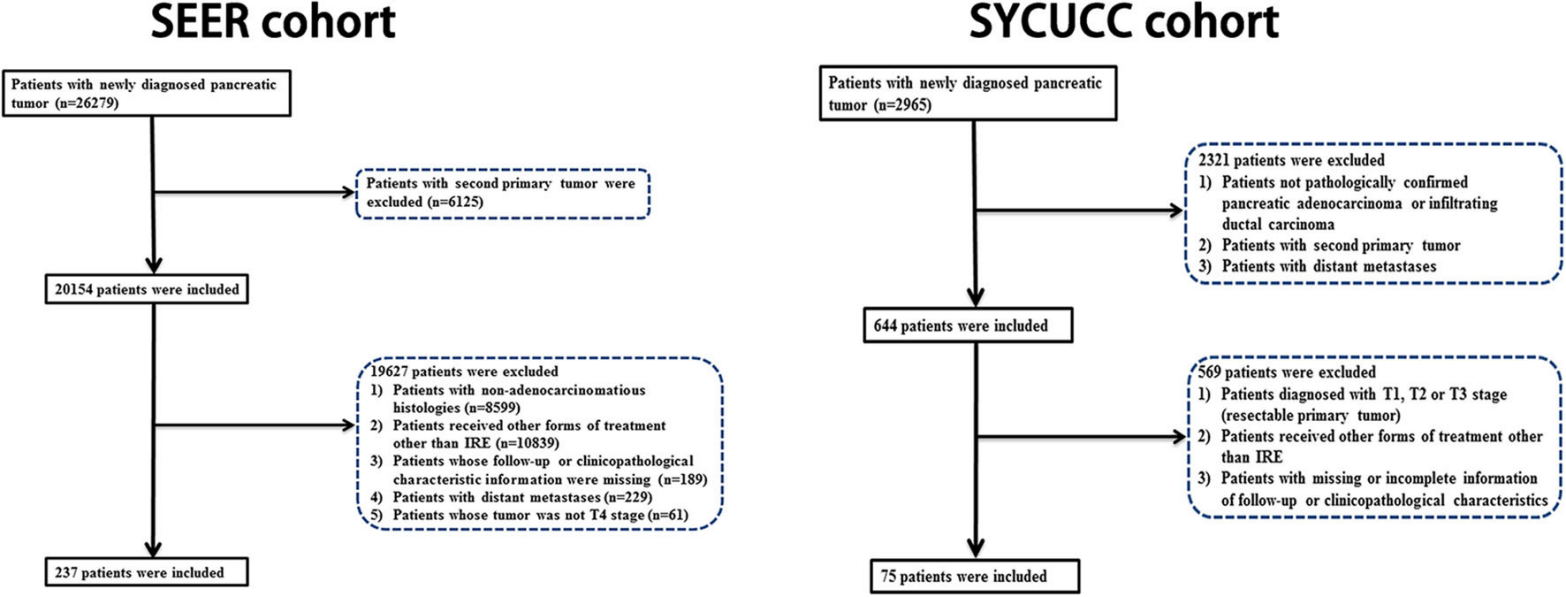
Flow diagram of the data selection process.

We identified 237 eligible LAPC patients who had received IRE treatment from the SEER database. In addition, consecutive LAPC patients who were initially treated with IRE between 2015 and 2019 at the Department of Pancreatobiliary Surgery of SYSUCC were also enrolled in this study. The flow diagram of the data selection is shown in Figure 1. There were 158 patients in the training cohort and 79 patients in the internal validation cohort. Another 75 patients from the SYSUCC database were used as an external validation cohort. The included patients had a median age of 66 years (range 26–93 years). Female patients (123, 51.9%) were a little more than male patients (114, 48.1%). The head of pancreas (70.8%) was the most common tumor site, followed by the tail (13.9%), the body (10.1%), and the overlapping sites of the pancreas (5.2%). A majority of patients (154, 49.7%) had tumors which were poorly differentiated. As for tumor size, the large size (lager than 4 cm, 45.6%) was the most common, followed by median size (2–4 cm, 43.9%) and small size (smaller than 2 cm, 10.5%). More than half of patients had received chemotherapy in both training and validation cohorts (Table 1).

**Table 1:** The comparison of clinicopathological factors between training cohort and validation cohort.

**Characteristic**		***N***	**Patients**		***P***
			**Training cohort**	**Validation cohort**	
Total		237	158	79	
Age (years)	<70	137	93	44	0.677
	≥70	100	65	35	
Gender	Male	114	79	35	0.491
	Female	123	79	44	
Chemotherapy	No	101	62	39	0.164
	Yes	136	96	40	
Tumor site	Head	168	113	55	0.491
	Body	24	18	6	
	Tail	33	21	12	
	Overlapping sites	12	6	6	
Tumor size (cm)	≤2	24	17	7	0.228
	2–4	104	63	41	
	>4	108	77	31	
Tumor grade	Well	17	11	6	0.928
	Moderate	66	43	23	
	Poor	154	104	50	
Survival status	Survival	14	11	3	0.385
	Cancer-specific mortality	170	115	55	
	Non-cancer-specific mortality	53	32	21	

**Figure 2:**
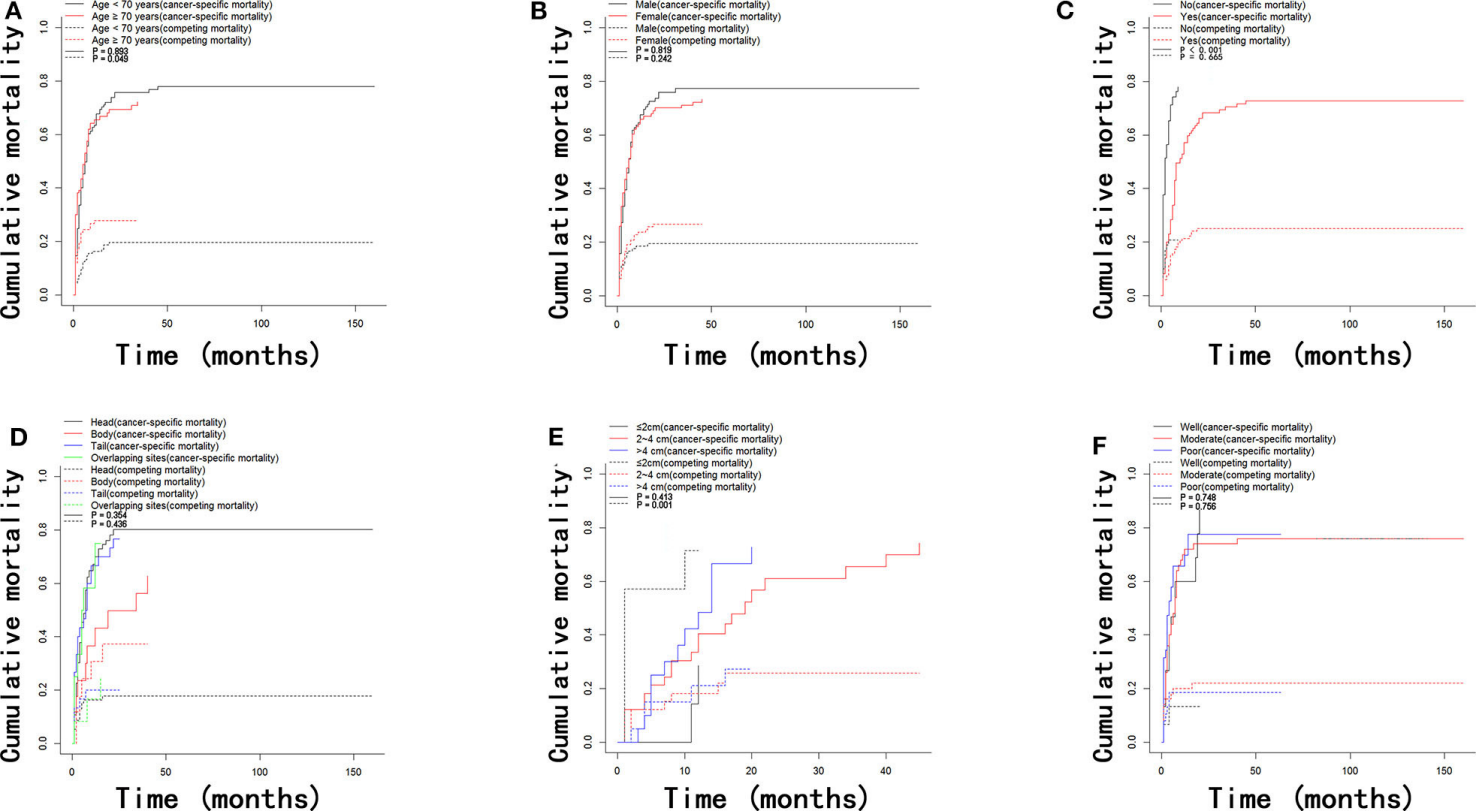
Cumulative mortality curves regarding cancer-specific and competing mortality stratified by patient characteristics: **(A)** age; **(B)** gender; **(C)** chemotherapy; **(D)** tumor site; **(E)** tumor size; **(F)** tumor grade.

In the present study, there were 170 deaths due to LAPC and 53 deaths due to other causes during the follow-up period with 8 months (range, 1–186 months) as the median time. The comparison of 1-, 2-, and 3-year OS rates, cancer-specific mortalities, and non-cancer-specific mortalities is summarized in Table 2. The corresponding CIF curves according to different features are shown in Figure 2. In the whole group, the 1-, 2-, and 3-year cumulative incidences of all-cause death were 66.7, 73.0, and 74.3%, respectively. There were no significant differences in cancer-specific and non-cancer-specific mortalities between male and female patients. Patients of older ages had significantly higher non-cancer-specific mortalities compared with the younger ones while the cancer-specific mortalities were comparable between these two groups. Chemotherapy contributed to significantly decreased mortality while it did not show a closed relationship with non-cancer-specific mortality. Compared with well-differentiated disease, patients with moderately or poorly differentiated tumors did not have significantly higher cancer-specific mortalities. Tumor site and tumor size did not predict the probability of cancer-specific mortality in LAPC patients after IRE treatment. Other than age, patients whose tumor was smaller than 2 cm had significant higher non-cancer-specific mortality (Table 2).

**Table 2:** Overall survival rates and cumulative incidences of mortality among patients with LAPC after IRE treatment.

**Characteristic**		**Patients**		**Overall survival rate (%)**			***P***	**Cancer-specific mortality (%)**			***P***	**Non-cancer-specific mortality (%)**			***P***
		**No**.	**%**	**1–year**	**2–year**	**3–year**		**1–year**	**2–year**	**3–year**		**1–year**	**2–year**	**3-year**
Total		237	100	12.2	3.8	2.5		66.7	73.0	74.3		21.1	23.2	23.2	
Age (years)	<70	137	58	16.0	4.6	4.6	<0.001	67.7	75.7	75.7	0.893	16.2	19.7	19.7	0.049
	≥70	100	42	6.8	2.7	0		65.4	69.5	NA		27.8	27.8	NA	
Gender	Male	114	48	14.0	4.6	3.0	0.247	67.5	75.9	77.4	0.819	18.5	19.5	19.5	0.242
	Female	123	52	10.4	3.1	2.1		65.9	70.1	71.2		23.6	26.8	26.8	
Chemotherapy	No	101	43	0	0	0	<0.001	NA	NA	NA	<0.001	NA	NA	NA	0.665
	Yes	136	57	21.5	6.7	2.06		57.1	68.2	70.5		21.3	25.0	25.0	
Tumor site	Head	168	71	13.8	2.0	2.0	0.504	70.0	80.2	80.2	0.354	16.2	17.8	17.8	0.436
	Body	24	10	26.1	13.1	6.5		43.1	49.7	56.2		30.7	37.3	37.3	
	Tail	33	14	13.3	3.3	3.3		66.7	76.7	NA		20.0	20.0	NA	
	Overlapping sites	12	5	8.3	0	0		75.0	NA	NA		16.7	NA	NA	
Tumor size (cm)	≤2	24	10	41.4	13.2	8.8	0.025	28.6	NA	NA	0.413	71.4	NA	NA	0.001
	2–4	104	44	30.6	0	0		40.4	61.1	65.5		18.2	25.7	25.7	
	>4	108	46	0	0	0		48.3	NA	NA		21.1	NA	NA	
Tumor grade	Well	17	7	26.7	0	0	0.436	60.0	NA	NA	0.748	13.3	NA	NA	0.756
	Moderate	66	28	8.0	4.0	2.0		72.0	74.0	74.0		20.0	22.0	22.0	
	Poor	154	65	11.8	4.0	4.0		69.7	77.6	77.6		18.4	18.4	18.4	

*LAPC, locally advanced pancreatic cancer; IRE, irreversible electroporation; NA, not available*.

### OS and CSS of Patients

**Figure 3:**
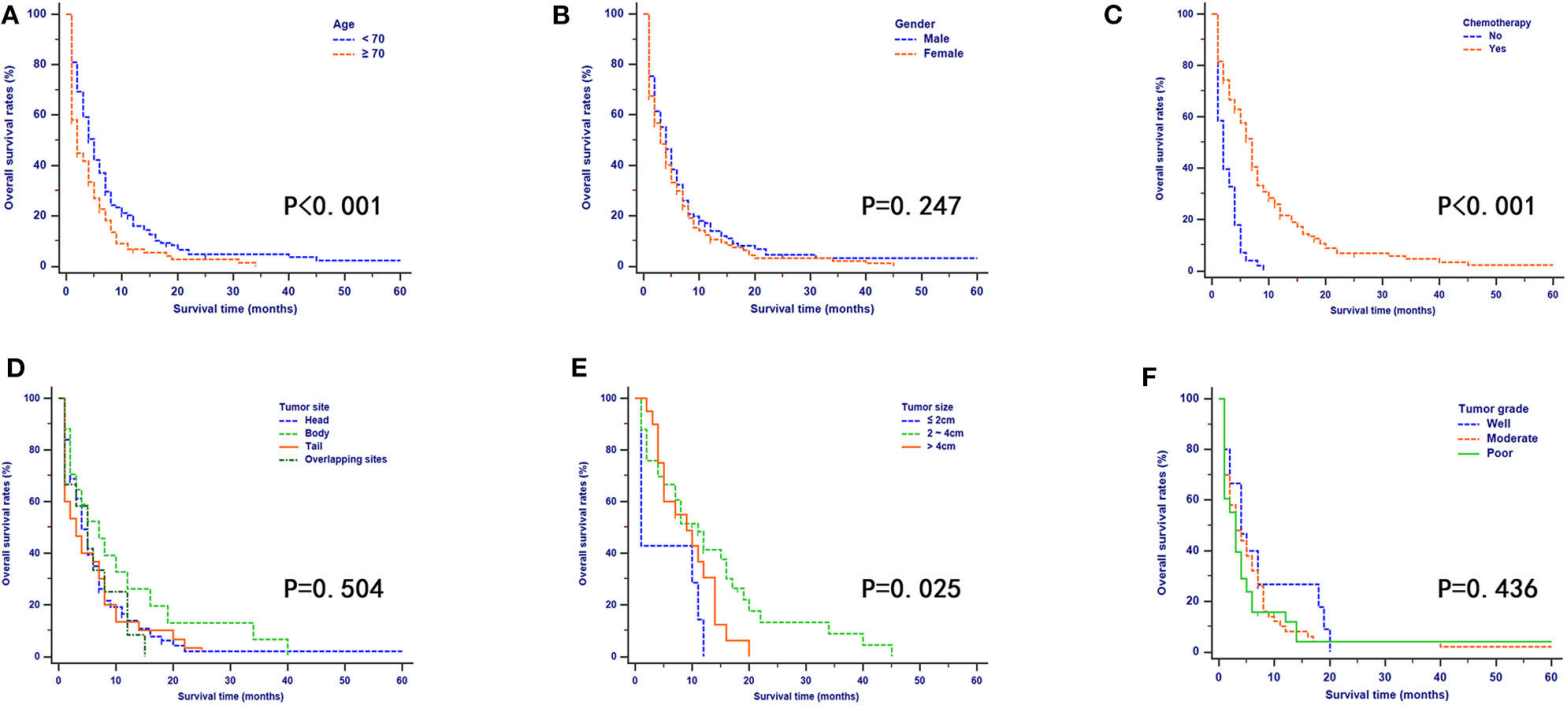
Kaplan–Meier OS curves stratified by patient characteristics: **(A)** age; **(B)** gender; **(C)** chemotherapy; **(D)** tumor site; **(E)** tumor size; **(F)** tumor grade.

According to the results of the OS analyses which are shown in Figure 3, OS rates of patients were significantly different when they were stratified by age, tumor size, and chemotherapy. There were no obvious differences in OS rates of patients when they were stratified by gender, tumor site, and tumor grade. Moreover, the univariate analysis revealed that age, tumor site, tumor size, tumor grade, and chemotherapy were all significantly associated with OS. These variables were then analyzed by multivariate analysis to delineate various prognostic indicators. It was shown that age (HR = 1.226, 95% CI, 1.101–1.987, *P* = 0.042), tumor site (HR = 1.647, 95% CI, 1.124–2.145, *P* = 0.023), tumor size (HR = 2.337, 95% CI, 1.684–3.114, *P* = 0.004), tumor grade (HR = 1.877, 95% CI, 1.442–2.482, *P* = 0.014), and chemotherapy (HR = 0.273, 95% CI, 0.121–0.367, *P* = 0.001) could strongly predict OS. Proportional subdistribution hazard assumption was held for variables used for CSS analysis. Age, tumor size, tumor grade, and chemotherapy were all independently associated with CSS. Moreover, age, tumor site, and tumor size were proved to be significantly related to NCSS (Table 3).

**Table 3:** Univariate and multivariate analyses of survival in patients with PC after IRE treatment.

**Characteristic**	**Overall survival**						**Cancer-specific survival**						**Non-cancer-specific survival**					
	**Univariate analysis**			**Multivariate analysis**			**Univariate analysis**			**Multivariate analysis**			**Univariate analysis**			**Multivariate analysis**		
	**HR**	**95% CI**	***P***	**HR**	**95% CI**	***P***	**HR**	**95% CI**	***P***	**HR**	**95% CI**	***P***	**HR**	**95% CI**	***P***	**HR**	**95% CI**	***P***
Age (years)			0.003			0.042			0.047			0.042			0.011			0.002
<70/≥70	1.510	1.153–1.977		1.226	1.101–1.987		1.371	1.004–1.873		1.124	1.002–1.568		2.031	1.178–3.053		2.113	1.486–4.525	
Gender			0.296			NI			0.680			NI			0.163			NI
Male/female	1.151	0.884–1.498					1.066	0.788–1.441					1.477	0.854–2.553				
Chemotherapy			0.001			0.001			0.001			0.001			0.187			NI
No/yes	0.285	0.210–0.386		0.273	0.121–0.367		0.243	0.170–0.346		0.223	0.143–0.355		0.457	0.248–1.142				
Tumor site			0.034			0.023			0.045			0.425			0.049			0.026
Head/body/tail/overlapping site	1.086	1.006–1.172		1.647	1.124–2.145		1.061	1.001–1.158		1.124	0.748–1.232		1.170	1.001–1.368		1.416	1.043–1.921	
Tumor size (cm)			0.002			0.004			0.012			0.002			0.002			0.009
≤ 2/2–4/>4	2.564	1.754–3.874		2.337	1.684–3.114		2.547	1.625–3.335		2.744	1.562–3.587		0.177	0.060–0.523		0.230	0.076–0.694	
Tumor grade						0.014			0.002			0.027			0.010			0.089
Well/moderate/poor	1.614	1.271–2.049	0.001	1.877	1.442–2.482		1.515	1.159–1.980		1.711	1.121–3.152		1.153	1.108–2.112		1.124	0.746–1.885	

*LN, lymph node; HR, hazard ratio; CI, confidence interval; NI, not included; other abbreviations as in Table 2*.

### Construction and Validation of Nomograms

**Figure 4:**
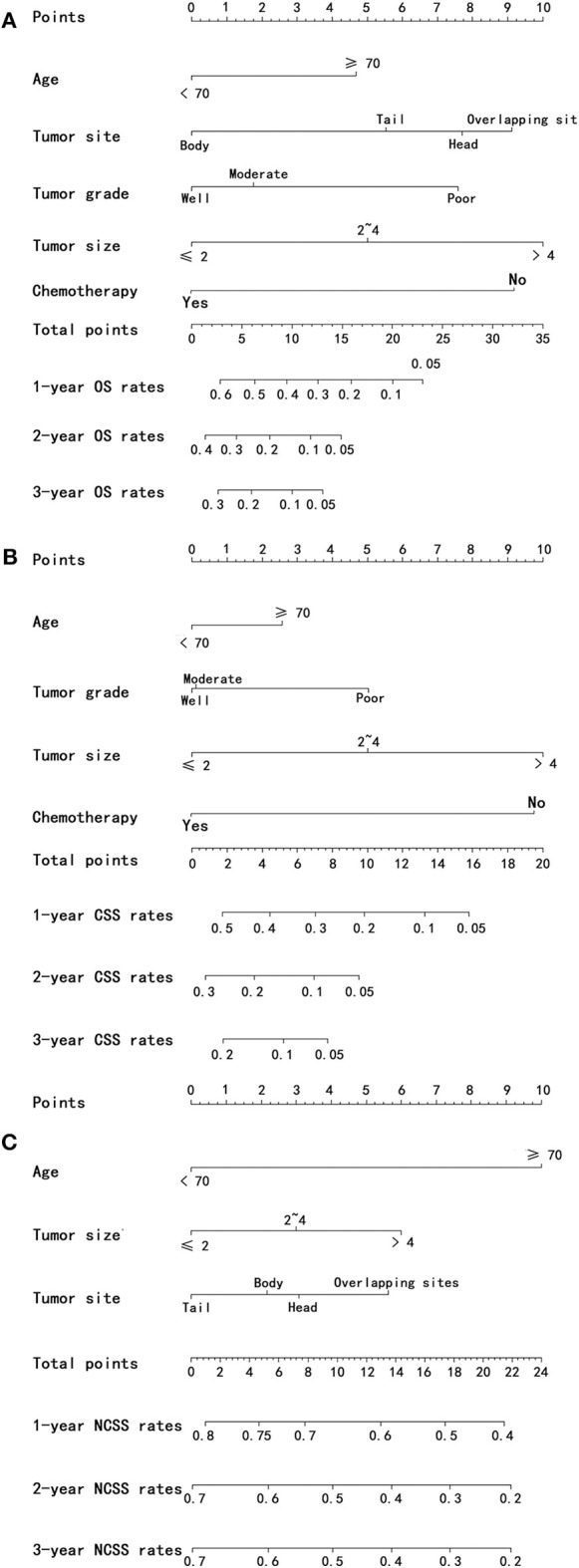
Nomograms predicting 1-, 2-, and 3-year OS **(A)**, CSS **(B)**, and NCSS **(C)** of patients with PC after IRE treatment. OS, overall survival; CSS, cancer-specific survival; NCSS, non-cancer-specific survival; PC, pancreatic cancer; IRE, irreversible electroporation.

**Figure 5:**
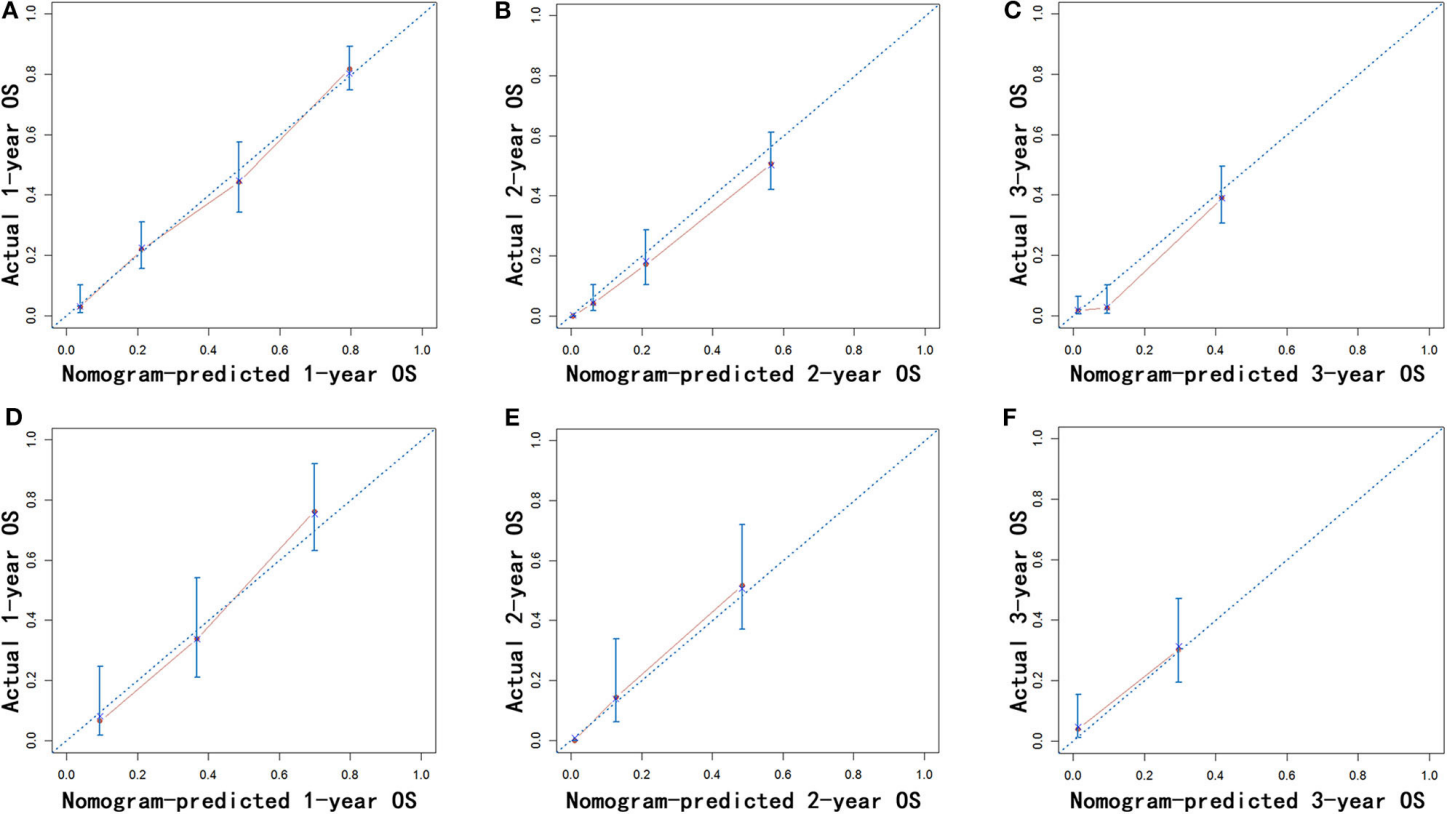
Calibration of the nomogram using the training **(A–C)** and validation cohorts **(D–F)** is shown. The x-axis represents the nomogram-predicted OS rate, and the y-axis represents the actual OS rate. OS, overall survival.

**Figure 6:**
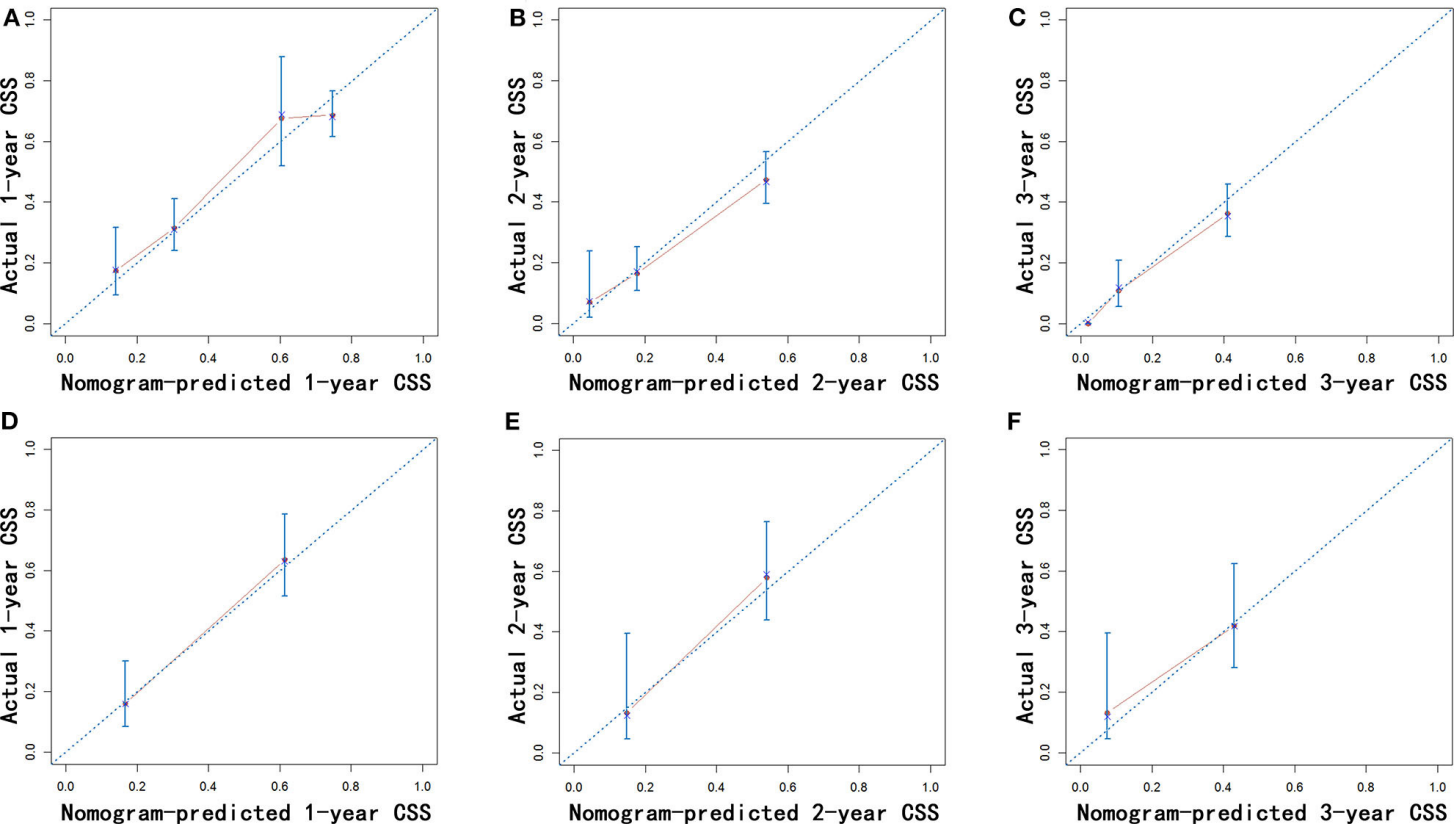
Calibration of the nomogram using the training **(A–C)** and validation cohorts **(D–F)** is shown. The x-axis represents the nomogram-predicted CSS rate, and the y-axis represents the actual CSS rate. CSS, cancer-specific survival.

**Figure 7:**
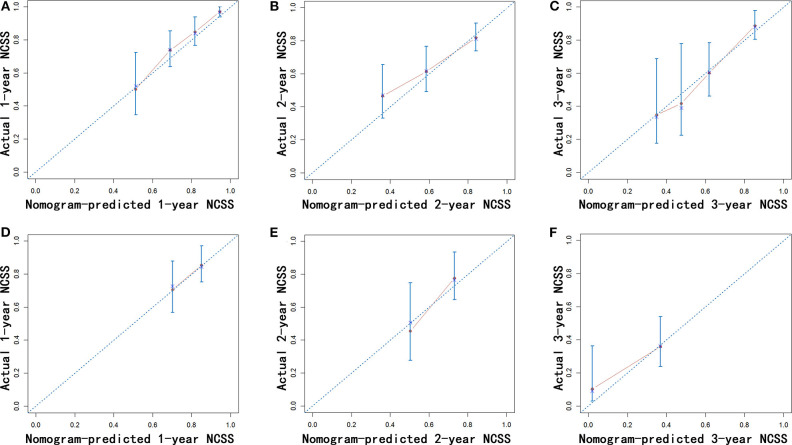
Calibration of the nomogram using the training **(A–C)** and validation cohorts **(D–F)** is shown. The x-axis represents the nomogram-predicted NCSS rate, and the y-axis represents the actual NCSS rate. NCSS, non-cancer-specific survival.

Nomograms were established with the independent predictors of OS, CSS, and NCSS (Figure 4). By adding the scores for each selected variables, the established nomograms can be used to predict the probability of 1-, 2-, and 3-year OS, CSS, and NCSS for patients with LAPC after IRE treatment. The well-calibrated curves for nomograms were observed in both training and internal validation cohorts (Figures 5–7). The C-index of nomogram for OS prediction was 0.782 (95% CI, 0.759–0.806). The nomogram for CSS and NCSS prediction showed great predictive power with C-indexes of 0.729 (95% CI, 0.696–0.762) and 0.730 (95% CI, 0.679–0.781), respectively. In addition, compared with the 7th or 8th edition TNM stage system, the established nomograms showed higher values of C-indexes, indicating enhanced discriminatory ability in predicting OS, CSS, and NCSS (Table 4). To further illustrate the clinical use of the established nomograms, the nomograms were validated in the SYSUCC cohort, which was used as an external cohort. The C-indexes of the external cohort were 0.780 (95% CI, 0.723–0.837) for OS and 0.776 (95% CI, 0.700–0.852) for CSS, respectively, which were both higher than those of the 7th or 8th edition TNM stage system (Table 4).

**Table 4:** C-indexes for the nomograms and TNM staging systems in patients with PC after IRE treatment.

**Survival**		**Training cohort**	***P***	**Internal validation cohort**	***P***	**External validation cohort**	***P***
Overall survival	Nomogram	0.782 (0.759–0.806)	Reference	0.742 (0.683–0.801)	Reference	0.780 (0.723–0.837)	Reference
	TNM 7th stage	0.490 (0.304–0.676)	<0.001	0.614 (0.566–0.662)	<0.001	0.528 (0.447–0.609)	<0.001
	TNM 8th stage	0.604 (0.492–0.716)	0.021	0.659 (0.614–0.704)	<0.001	0.530 (0.432–0.628)	<0.001
Cancer-specific survival	Nomogram	0.729 (0.696–0.762)	Reference	0.737 (0.676–0.800)	Reference	0.776 (0.701–0.852)	Reference
	TNM 7th stage	0.506 (0.390–0.622)	0.002	0.627 (0.573–0.681)	<0.001	0.559(0.476–0.642)	<0.001
	TNM 8th stage	0.636 (0.530–0.742)	0.198	0.666 (0.613–0.719)	<0.001	0.569(0.470–0.668)	<0.001
Non-cancer-specific survival	Nomogram	0.730 (0.679–0.781)	Reference	0.643 (0.516–0.770)	Reference	NA	NA
	TNM 7th stage	0.545 (0.377–0.713)	<0.001	0.622 (0.539–0.705)	0.008	NA	NA
	TNM 8th stage	0.481 (0.242–0.720)	<0.001	0.564 (0.473–0.655)	<0.001	NA	NA

*Abbreviations as in Table 2, NA, not available*.

**Figure 8:**
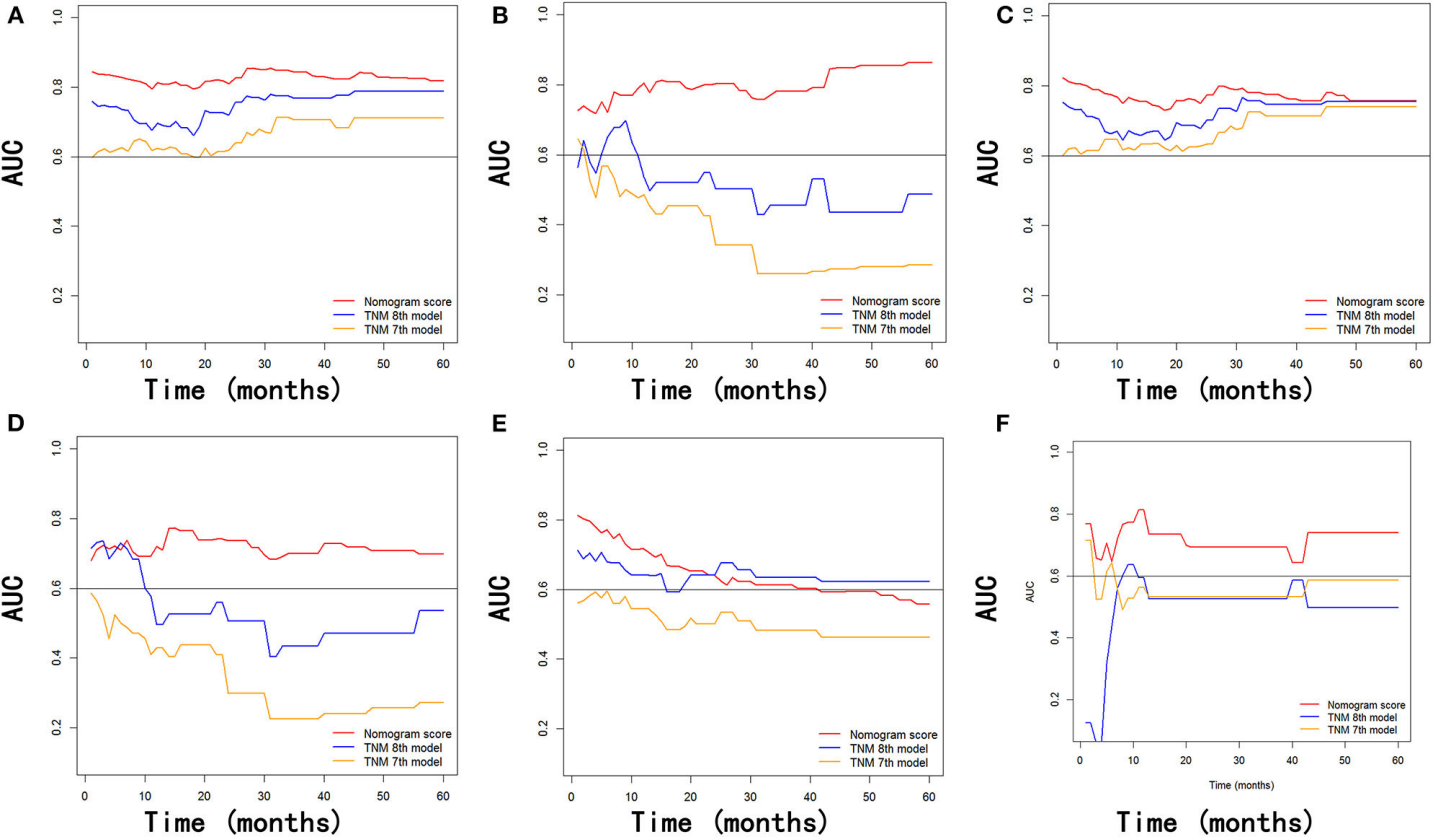
Comparison of the values of AUC of the nomogram and the TNM staging systems for 1-, 2-, and 3-year OS prediction **(A,B)**, CSS prediction **(C,D)**, and NCSS prediction **(E,F)** in the training and validation cohorts. AUC, area under ROC curves; OS, overall survival; CSS, cancer-specific survival; NCSS, non-cancer-specific survival.

In addition, the comparison of AUC values of the stage systems is shown in Figure 8. The AUC values of the nomograms for predicting 1-, 2-, and 3-year OS and CSS were 0.720, 0.720, and 0.768 and 0.720, 0.717, and 0.774, respectively, in the training cohort, which were higher than those of 7th and 8th TNM stage systems. Also, the AUC values of the nomograms on the external validation cohort for predicting 1-, 2-, and 3-year survival were 0.732 and 0.830, 0.756 and 0.762, and 0.698 and 0.696 for OS and CSS, respectively, which were all highest among those of different stage systems. Moreover, the established nomograms also showed superior discriminatory capacity in predicting NCSS in both training and validation cohorts (Table 5).

**Table 5:** Values of AUC for the nomograms and TNM staging systems in patients with PC after IRE treatment.

**Survival**		**Training cohort**			**Internal validation cohort**			**External validation cohort**		
		**1–year**	**2–year**	**3–year**	**1–year**	**2–year**	**3–year**	**1–year**	**2–year**	**3–year**
Overall survival	Nomogram	0.720	0.720	0.768	0.683	0.692	0.670	0.732	0.756	0.698
	TNM 7th stage	0.637	0.649	0.712	0.523	0.305	0.201	0.471	0.732	0.528
	TNM 8th stage	0.676	0.701	0.773	0.598	0.596	0.501	0.589	0.681	0.638
Cancer-specific survival	Nomogram	0.720	0.717	0.774	0.623	0.682	0.648	0.830	0.762	0.696
	TNM 7th stage	0.637	0.645	0.710	0.483	0.315	0.208	0.477	0.754	0.538
	TNM 8th stage	0.674	0.589	0.775	0.512	0.567	0.454	0.663	0.707	0.646
Non-cancer-specific survival	Nomogram	0.624	0.583	0.526	0.704	0.573	0.573	NA	NA	NA
	TNM 7th stage	0.579	0.560	0.560	0.597	0.446	0.446	NA	NA	NA
	TNM 8th stage	0.615	0.608	0.614	0.681	0.561	0.561	NA	NA	NA

*AUC, area under ROC curve; other abbreviations as in Table 4*.

## Discussion

LAPC has a steadily increasing incidence rate and aggressive nature. The resectable rate at diagnosis is relatively low, and the involvement of the nearby vascular structures contributes to 30% of unresectable tumors (5). IRE is a novel method and has been proven to be an effective treatment for LAPC (16). Due to the variation of clinical and pathological features of LAPC patients after IRE treatment, it is imprecise to estimate prognosis for these patients relying on the traditional stage systems. It is necessary to evaluate the prognostic factors and establish an efficient stage system which is specially for these patients. Moreover, many patients were diagnosed at their older ages and the increasing ages might have an impact on their survival. However, previous stage systems only considered OS, ignoring to evaluate the prognostic impact of age (24–26). Thus, in this study, we sought to evaluate the demographic, clinical, and pathological features of LAPC patients after IRE treatment and establish prognostic nomograms to predict OS, CSS, and NCSS.

Similar to other studies (27, 28), most patients were nearly 70 years old when they were diagnosed with LAPC. It was observed that age was associated with OS, CSS, and NCSS. This means that older patients are at great risks of cancer-specific death and non-cancer-specific death. In addition, it was shown that the increasing age was proven to be an independent risk factor for long-term survival in this study, especially NCSS, for which age had displayed the greatest prognostic impact. This result was in agreement with that from other studies (29, 30). The age-related comorbid conditions played an important role in non-cancer-specific mortality. With this, it was a suitable method to take surgery tolerance into account to evaluate prognosis of LAPC patients who had received IRE treatment.

In the presence of the competing risk model, apart from age, other factors which were shown to independently predict OS and CSS included tumor site, tumor grade, tumor size, and chemotherapy. Involvement of the vascular structure and tumor size was the predominant feature of the included patients in this study. Gray's model also consolidated the determinant role of tumor size in predicting CSS in this study. In addition, our study showed that, compared with OS, tumor size weighted more in predicting CSS which was mainly influenced by the inherent characteristics of tumor itself. Tumor grade and tumor site were also proven to be prognostic factors and were included in the stage system in this study, which was in accordance with other similar studies (27, 31). Our analysis supported the prognostic significance of tumor site in predicting OS and indicated that LAPC patients occurring in the head or overlapping sites of the pancreas had a decreasing probability of OS compared with diseases occurring in the body and tail of pancreas. Moreover, tumor grade and tumor site have also displayed prognostic values, which was independent of other important prognostic factors from the TNM stage system in this study. As for chemotherapy combined with IRE, in addition to local disease control by IRE, chemotherapy could adequately control microscopic diseases. Additionally, IRE could also assist chemotherapy delivery to the tumor by disrupting the dense stroma of PC (32, 33). Therefore, the synergistic effect from the combination of chemotherapy and IRE could contribute to a significantly prolonged survival.

Patient counseling and decision-making are based on the prognosis estimated from the individual risk profiles. With the increasing concern for non-cancer-specific mortality, competing risk analyses have been adopted in more and more cancer researches, such as lung cancer, breast cancer, gastric cancer, and head and neck cancer (30, 34–36). Considering that 23.8% of deaths were caused by competing causes other than primary LAPC, the competing interesting causes were taken into account when the prognosis is evaluated in our study. As far as we know, it was the first time to evaluate the prognostic factors based on the competing risk analysis model for LAPC patients after IRE treatment. Apart from the factors included in the TNM stage system, age, was generally accepted as a determinant of comorbidity, and tumor grade and chemotherapy were both integrated into the present-stage system. The established nomograms displayed higher C-indexes and values of AUC, indicating better discriminatory power in predicting OS, CSS, and NCSS. To further illustrate the clinical use of the established nomograms, the nomograms were validated in the SYSUCC cohort, which was used as an external cohort. The C-indexes of the external cohort were significantly higher than those of the 7th or 8th edition TNM stage system. That is to say, the well-validated nomograms can be used to predict survival of LAPC patients in clinical practice.

The superior power of nomograms in predicting survival may partly be due to the inclusion of additional variables. Moreover, the results of our research which were based on the analyses from a relatively large population-based database were more generable than those from single-center studies. The present study was therefore the first to evaluate prognostic factors based on large cohorts for LAPC patients after IRE. The nomograms, which comprise a few easily obtained predictors, could help doctors make accurate individual prognosis estimates and select groups of patients with different risks of decreased survival after IRE. Patients with high risks of decreased survival, which were suggested by survival estimation of nomograms, could benefit more from adjuvant therapies, including chemotherapy and radiotherapy. Therefore, with this easily used predictive system, diverse risk factors of patients could be assessed by doctors more objectively and precisely. However, rigorous tests and validations with more external cohorts, specially study cohorts from perspective studies, are needed for the established nomograms before they are formally adopted in clinical practice. Finally, a more optimized prognosis estimation would contribute to more specialized personal treatment.

There are some limitations for this study. First, the nomograms were generated from baseline characteristics of LAPC patients. The addition of some potential prognostic variables, such as carbohydrate antigen 19-9, lymph node metastasis, and vascular invasion, which were unavailable in the SEER dataset, may further improve the predictive power of the present-stage system. Second, the nomograms were generated from patients after specific treatment (IRE). They were not suitable for all LAPC patients. Third, the development of chemotherapy would cause the differences and changes in regions or causes along with time. Therefore, the adjustment and perfection of the chemotherapy variables in the nomograms are an important work to do in the future. Although the nomograms were generated from a large population-based database and validated in an external cohort, further wide validation based on other population is still needed to estimate the accuracy of models.

In conclusion, we evaluated cancer-specific and non-cancer-specific deaths in LAPC patients after IRE treatment and established nomograms to specially predict OS, CSS, and NCSS for these patients. The established nomograms exhibited relatively good performance in predicting survival and might facilitate highly tailored patient management in clinical practice.

## Data Availability Statement

The datasets generated for this study are available on request to the corresponding author.

## Ethics Statement

The studies involving human participants were reviewed and approved by Institutional Review Board of Sun Yat-sen University Cancer Center. The patients/participants provided their written informed consent to participate in this study.

## Author Contributions

SL was responsible for conception, design, quality control of this study, reviewed and edited the manuscript respectively. CH, XH, and YZ performed the study selection, data extraction, statistical analyses, and was major contributors in writing the manuscript, participated in studies selection and statistical analyses, and contributed to the writing of manuscript. CH, XH, YZ, and XL contributed in classification criteria discussion. All authors contributed to the article and approved the submitted version.

## Conflict of Interest

The authors declare that the research was conducted in the absence of any commercial or financial relationships that could be construed as a potential conflict of interest.

## References

[1] Siegel RL Miller KD Jemal A Cancer statistics, 2016. CA Cancer J Clin. (2016) 66:7–30. 10.3322/caac.21332 26742998

[2] Siegel RL Miller KD Jemal A Cancer statistics, 2018. CA Cancer J Clin. (2018) 68:7–30. 10.3322/caac.21442 29313949

[3] Hidalgo M. Pancreatic cancer. N Eng J Med. (2010) 362:1605–17. 10.1056/NEJMra0901557 20427809

[4] Hartwig W Hackert T Hinz U Gluth A Bergmann F Strobel O. Pancreatic cancer surgery in the new millennium: better prediction of outcome. Ann Surg. (2011) 254:311–9. 10.1097/SLA.0b013e31821fd334 21606835

[5] Weiss MJ Wolfgang CL. Irreversible electroporation: a novel therapy for stage III pancreatic cancer. Adv Surg. (2014) 48:253–8. 10.1016/j.yasu.2014.05.002 25293620

[6] Lafranceschina S Brunetti O Delvecchio A Conticchio M Ammendola M Curro G. Systematic review of irreversible electroporation role in management of locally advanced pancreatic cancer. Cancers. (2019) 11:1718. 10.3390/cancers11111718 31684186 PMC6896066

[7] Paiella S De Pastena M D'Onofrio M Crino SF Pan TL De Robertis R. Palliative therapy in pancreatic cancer-interventional treatment with radiofrequency ablation/irreversible electroporation. Transl Gastroenterol Hepatol. (2018) 3:80. 10.21037/tgh.2018.10.05 30505967 PMC6232064

[8] Porcelli L Quatrale AE Mantuano P Leo MG Silvestris N Rolland JF. Optimize radiochemotherapy in pancreatic cancer: PARP inhibitors a new therapeutic opportunity. Mol Oncol. (2013) 7:308–22. 10.1016/j.molonc.2012.10.002 23148997 PMC5528465

[9] Martin RC II McFarland K Ellis S Velanovich V. Irreversible electroporation in locally advanced pancreatic cancer: potential improved overall survival. Ann Surg Oncol. (2013) 20 (Suppl. 3):S443–9. 10.1245/s10434-012-2736-1 23128941

[10] Cannon R Ellis S Hayes D Narayanan G Martin RC II. Safety and early efficacy of irreversible electroporation for hepatic tumors in proximity to vital structures. J Surg Oncol. (2013) 107:544–9. 10.1002/jso.23280 23090720

[11] Lin M Liang S Wang X Liang Y Zhang M Chen J. Percutaneous irreversible electroporation combined with allogeneic natural killer cell immunotherapy for patients with unresectable (stage III/IV) pancreatic cancer: a promising treatment. J Cancer Res Clin Oncol. (2017) 143:2607–18. 10.1007/s00432-017-2513-4 28871458 PMC11819031

[12] Martin RC II McFarland K Ellis S Velanovich V. Irreversible electroporation therapy in the management of locally advanced pancreatic adenocarcinoma. J Am Coll Surg. (2012) 215:361–9. 10.1016/j.jamcollsurg.2012.05.021 22726894

[13] Bower M Sherwood L Li Y Martin R. Irreversible electroporation of the pancreas: definitive local therapy without systemic effects. J Surg Oncol. (2011) 104:22–8. 10.1002/jso.21899 21360714

[14] Lee EW Wong D Prikhodko SV Perez A Tran C Loh CT. Electron microscopic demonstration and evaluation of irreversible electroporation-induced nanopores on hepatocyte membranes. J Vascul Interv Radiol. (2012) 23:107–13. 10.1016/j.jvir.2011.09.020 22137466

[15] Martin RC Philips P Ellis S Hayes D Bagla S. Irreversible electroporation of unresectable soft tissue tumors with vascular invasion: effective palliation. BMC Cancer. (2014) 14:540. 10.1186/1471-2407-14-540 25064086 PMC4124136

[16] Martin RC II Kwon D Chalikonda S Sellers M Kotz E Scoggins C. Treatment of 200 locally advanced (stage III) pancreatic adenocarcinoma patients with irreversible electroporation: safety and efficacy. Ann Surg. (2015) 262:486–94; discussion: 92–4. 10.1097/SLA.0000000000001441 26258317

[17] Amin MB ES Greene F AJCC Cancer Staging Manual. 8th ed Chicago, IL: Springer (2017).

[18] Muss HB Biganzoli L Sargent DJ Aapro M. Adjuvant therapy in the elderly: making the right decision. J Clin Oncol. (2007) 25:1870–5. 10.1200/JCO.2006.10.3457 17488985

[19] He C Wang J Zhang Y Lin X Li S. Irreversible electroporation after induction chemotherapy versus chemotherapy alone for patients with locally advanced pancreatic cancer: a propensity score matching analysis. Pancreatology. (2020) 20:477–84. 10.1016/j.pan.2020.02.009 32131993

[20] Fine JP Gray RJ A proportional hazards model for the subdistribution of a competing risk. J Am Stat Assoc. (1999) 94:496–509. 10.1080/01621459.1999.10474144

[21] Gray RJ A class of K-sample tests for comparing the cumulative incidence of a competing risk. Ann Stat. (1988) 16:1141–54. 10.1214/aos/1176350951

[22] Harrell FE Jr Lee KL Mark DB. Multivariable prognostic models: issues in developing models, evaluating assumptions and adequacy, and measuring and reducing errors. Stat Med. (1996) 15:361–87. 10.1002/(SICI)1097-0258(19960229)15:4<361::AID-SIM168>3.0.CO;2-4 8668867

[23] Pencina MJ D'Agostino RB. Overall C as a measure of discrimination in survival analysis: model specific population value and confidence interval estimation. Stat Med. (2004) 23:2109–23. 10.1002/sim.1802 15211606

[24] Adamu M Nitschke P Petrov P Rentsch A Distler M Reissfelder C. Validation of prognostic risk scores for patients undergoing resection for pancreatic cancer. Pancreatology. (2018) 18:585–91. 10.1016/j.pan.2018.05.005 29866508

[25] Shen YN Bai XL Jin G Zhang Q Lu JH Qin RY. A preoperative nomogram predicts prognosis of up front resectable patients with pancreatic head cancer and suspected venous invasion. HPB. (2018) 20:1034–43. 10.1016/j.hpb.2018.06.1956 29929784

[26] Hang J Wu L Zhu L Sun Z Wang G Pan J. Prediction of overall survival for metastatic pancreatic cancer: development and validation of a prognostic nomogram with data from open clinical trial and real-world study. Cancer Med. (2018) 7:2974–84. 10.1002/cam4.1573 29856121 PMC6051216

[27] He C Mao Y Wang J Duan F Lin X Li S. Nomograms predict long-term survival for patients with periampullary adenocarcinoma after pancreatoduodenectomy. BMC Cancer. (2018) 18:327. 10.1186/s12885-018-4240-x 29580215 PMC5870913

[28] Pu N Gao S Xu Y Zhao G Lv Y Nuerxiati A. Alkaline phosphatase-to-albumin ratio as a prognostic indicator in pancreatic ductal adenocarcinoma after curative resection. J Cancer. (2017) 8:3362–70. 10.7150/jca.20917 29158809 PMC5665053

[29] Eguchi T Bains S Lee MC Tan KS Hristov B Buitrago DH. Impact of increasing age on cause-specific mortality and morbidity in patients with stage I non-small-cell lung cancer: a competing risks analysis. J Clin Oncol. (2017) 35:281–90. 10.1200/JCO.2016.69.0834 28095268 PMC5456376

[30] Zhou H Zhang Y Qiu Z Chen G Hong S Chen X. Nomogram to predict cause-specific mortality in patients with surgically resected stage I non-small-cell lung cancer: a competing risk analysis. Clin Lung Cancer. (2018) 19:e195–203. 10.1016/j.cllc.2017.10.016 29153966

[31] He C Mao Y Wang J Huang X Lin X Li S. Surgical management of periampullary adenocarcinoma: defining an optimal prognostic lymph node stratification schema. J Cancer. (2018) 9:1667–79. 10.7150/jca.24109 29760806 PMC5950597

[32] Moir J White SA French JJ Littler P Manas DM. Systematic review of irreversible electroporation in the treatment of advanced pancreatic cancer. Eur J Surg Oncol. (2014) 40:1598–604. 10.1016/j.ejso.2014.08.480 25307210

[33] Narayanan G Hosein PJ Arora G Barbery KJ Froud T Livingstone AS. Percutaneous irreversible electroporation for downstaging and control of unresectable pancreatic adenocarcinoma. J Vascul Interv Radiol. (2012) 23:1613–21. 10.1016/j.jvir.2012.09.012 23177107

[34] Lian M Perez M Liu Y Schootman M Frisse A Foldes E. Neighborhood socioeconomic deprivation, tumor subtypes, and causes of death after non-metastatic invasive breast cancer diagnosis: a multilevel competing-risk analysis. Breast Cancer Res Treat. (2014) 147:661–70. 10.1007/s10549-014-3135-z 25234843 PMC4181526

[35] Shin DW Suh B Park Y Lim H Suh YS Yun JM. Risk of coronary heart disease and ischemic stroke incidence in gastric cancer survivors: a nationwide study in Korea. Ann Surg Oncol. (2018) 25:3248–56. 10.1245/s10434-018-6635-y 30043317

[36] Shen W Sakamoto N Yang L. Cancer-specific mortality and competing mortality in patients with head and neck squamous cell carcinoma: a competing risk analysis. Ann Surg Oncol. (2015) 22:264–71. 10.1245/s10434-014-3951-8 25074664

